# ADAM17 Confers Temozolomide Resistance in Human Glioblastoma Cells and miR-145 Regulates Its Expression

**DOI:** 10.3390/ijms24097703

**Published:** 2023-04-22

**Authors:** Jen-Tsung Yang, I-Neng Lee, Cheng Huang, Hsiu-Chen Huang, Yu-Ping Wu, Zhi-Yong Chong, Jui-Chieh Chen

**Affiliations:** 1 Department of Neurosurgery, Chang Gung Memorial Hospital, Chiayi Branch, Chiayi 61363, Taiwan; jents716815@yahoo.com.tw; 2 School of Traditional Chinese Medicine, College of Medicine, Chang Gung University, Taoyuan 33302, Taiwan; 3 Department of Medical Research, Chang Gung Memorial Hospital, Chiayi 61363, Taiwan; geneasylum@yahoo.com.tw (I.-N.L.);; 4 Department of Biotechnology and Laboratory Science in Medicine, National Yang Ming Chiao Tung University, Taipei 11221, Taiwan; chengh@ym.edu.tw; 5 Department of Applied Science, National Tsing Hua University South Campus, Hsinchu 30013, Taiwan; jane@mail.nhcue.edu.tw; 6 Center for Teacher Education, National Tsing Hua University, Hsinchu 300044, Taiwan; 7 Department of Biochemical Science and Technology, National Chiayi University, Chiayi 60004, Taiwan

**Keywords:** glioblastoma multiforme (GBM), temozolomide (TMZ), micro-RNAs (miRNAs), a disintegrin and metalloproteinase-17 (ADAM17), drug resistance

## Abstract

Glioblastoma (GBM) is a malignant brain tumor, commonly treated with temozolomide (TMZ). Upregulation of A disintegrin and metalloproteinases (ADAMs) is correlated to malignancy; however, whether ADAMs modulate TMZ sensitivity in GBM cells remains unclear. To explore the role of ADAMs in TMZ resistance, we analyzed changes in ADAM expression following TMZ treatment using RNA sequencing and noted that ADAM17 was markedly upregulated. Hence, we established TMZ-resistant cell lines to elucidate the role of ADAM17. Furthermore, we evaluated the impact of ADAM17 knockdown on TMZ sensitivity in vitro and in vivo. Moreover, we predicted microRNAs upstream of ADAM17 and transfected miRNA mimics into cells to verify their effects on TMZ sensitivity. Additionally, the clinical significance of ADAM17 and miRNAs in GBM was analyzed. ADAM17 was upregulated in GBM cells under serum starvation and TMZ treatment and was overexpressed in TMZ-resistant cells. In in vitro and in vivo models, ADAM17 knockdown conferred greater TMZ sensitivity. miR-145 overexpression suppressed ADAM17 and sensitized cells to TMZ. ADAM17 upregulation and miR-145 downregulation in clinical specimens are associated with disease progression and poor prognosis. Thus, miR-145 enhances TMZ sensitivity by inhibiting ADAM17. These findings offer insights into the development of therapeutic approaches to overcome TMZ resistance.

## 1. Introduction

Gliomas, which are derived from glial cells, account for nearly 24% of all brain cancers and 80.9% of malignant tumors. Among gliomas, glioblastoma multiforme (GBM), also known as grade IV astrocytoma, is the most aggressive and lethal type of brain cancer, accounting for 59.2% of all gliomas [1]. Standard clinical management involves surgery combined with radiotherapy and chemotherapy. However, the median post-diagnosis survival duration of patients remains below 15 months [2,3]. Therefore, understanding the molecular mechanisms of GBM treatment will facilitate early diagnosis and improve patient survival.

Temozolomide (TMZ) is a DNA alkylating agent that readily penetrates the blood–brain barrier and alkylates the guanine O^6^ position of nucleic acids in the nucleus, leading to DNA damage and cell death [4]. Combining TMZ with other therapeutic approaches has been shown to improve the quality of life of patients and extend their survival duration [2]. Despite the documented anticancer activity of TMZ, the emergence of intrinsic or acquired resistance to treatment remains a prevalent issue, undermining its effectiveness and leading to treatment failure in many cases [5,6,7].

A disintegrin and metalloproteinase-17 (ADAM17) was initially identified as a TNF-α converting enzyme (TACE) due to its ability to cleave membrane-bound TNF-α, thereby activating EGFR signaling pathways [8,9]. However, the function of ADAM17 is not limited to promoting the release of soluble TNFα, and it can process a wide range of substrates, exceeding 70 in total, including ligands of the epidermal growth factor receptor (EGFR), cytokines, cytokine receptors, and adhesion molecules [10,11]. ADAM17 overexpression is correlated to more advanced tumor stages and negative outcome in patients with glioma [12]. Furthermore, ADAM17 inhibition in human GBM cell lines significantly inhibited cell invasion [13]. However, whether ADAM17 modulates TMZ sensitivity in GBM cells remains unclear. Hence, deeper understanding of the roles of ADAM17 in drug resistance may aid the development of novel therapeutic strategies for GBM in clinical settings, particularly regarding TMZ resistance.

MicroRNAs (miRNAs) are a class of endogenous non-coding RNAs comprising approximately 22 nucleotides. miRNAs regulate gene expression at the post-transcriptional level [14]. miRNA dysregulation has been observed in GBM, which may be involved in the pathogenesis, progression, and therapeutic resistance of this malignancy [15,16,17]. miR-145 is a tumor suppressor gene expressed in various tumors, including esophageal, ovarian, colon, bladder, and prostate cancers, and its expression levels in cancer tissues are lower than those in normal tissues [18,19,20,21,22]. While miR-145 can suppress ADAM17 expression in glioma cells, thereby inhibiting cell migration and invasion, whether miR-145 enhances TMZ sensitivity in GBM by regulating ADAM17 remains uncertain [13,23].

To this end, in the present study, we established a TMZ-resistant cell line to investigate the roles of ADAM17 and miR-145 in drug resistance. Next, we used genetic engineering to knock down ADAM17 or overexpress miR-145 to reduce ADAM17 protein expression and verified its effect on TMZ sensitivity through in vitro and in vivo experiments. Additionally, we used several online databases to analyze the clinical significance of ADAM17 and miR-145 in GBM. To the best of our knowledge, the present study is the first to report the link between ADAM17 expression and TMZ sensitivity in GBM. Our observations may serve as a foundation for the formulation of novel biomarkers and treatment plans to address TMZ resistance in patients with GBM.

## 2. Results

### 2.1. Serum Starvation and TMZ Treatment Enhanced ADAM17 Expression, Which May Contribute to the Development of TMZ Resistance in GBM Cells

**Figure 1:**
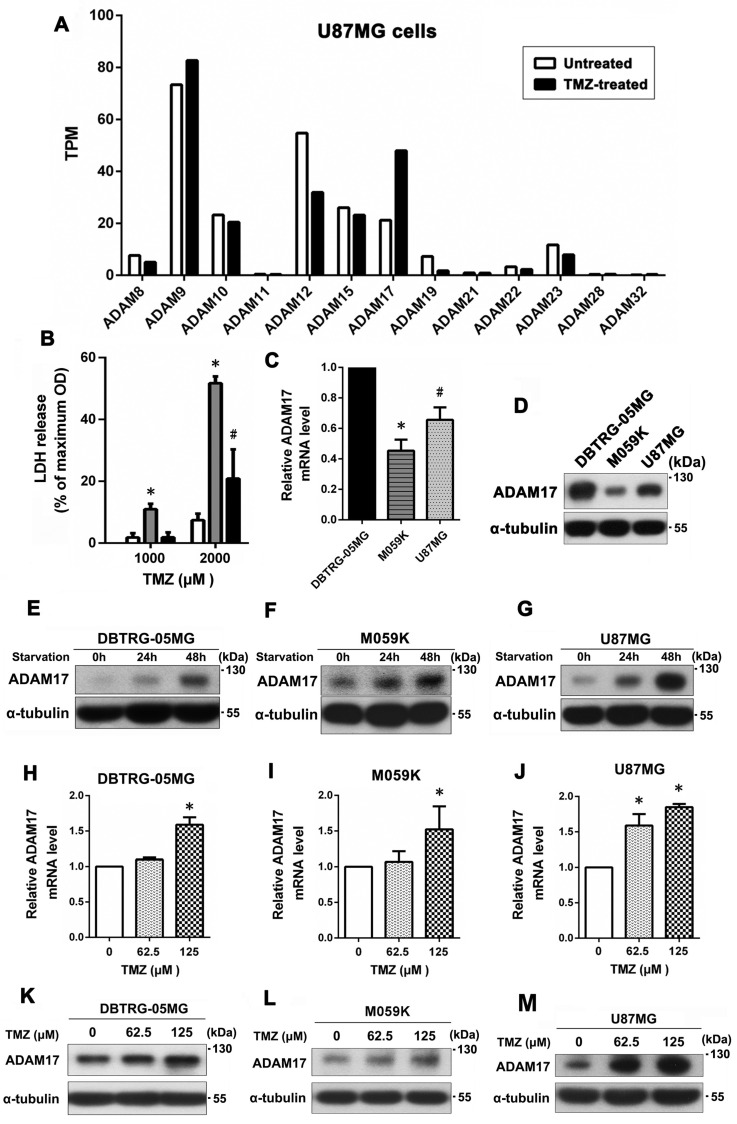
ADAM17 expression was induced under serum deprivation and TMZ treatment, which was related to TMZ resistance in GBM cell lines. (**A**) U87MG cells were treated or not with TMZ, and the relative gene expression levels (TPM) of 13 different ADAMs were measured using RNA sequencing data. (**B**) Three GBM cell lines were treated with 1000 or 2000 μM TMZ for 24 h, and cell death was evaluated using an LDH-based cytotoxicity assay. (**C**,**D**) Transcript and protein expression levels of ADAM17 in the three GBM cell lines were evaluated using q-PCR and Western blotting, respectively. * *p <* 0.05, compared with DBTRG-05MG and U87MG, ^#^ *p <* 0.05, compared with DBTRG-05MG and M059K. (**E**–**G**) Cells were cultured in a serum-withdrawal medium for 24 and 48 h, and ADAM17 expression in three GBM cell lines was analyzed by Western blotting. (**H**–**J**) Cells were treated with increasing doses of TMZ (0–125 μM) for 48 h, and the transcript expression of ADAM17 in the three GBM cell lines was quantitated using qPCR. * *p <* 0.05, compared with untreated controls. (**K**–**M**) Effect of TMZ on ADAM17 protein levels in GBM cells was analyzed using Western blotting.

Initially, we employed RNA sequencing to investigate the expression levels of ADAMs in U87MG cells following TMZ treatment and identify the specific members of the ADAMs protein family whose expression levels were altered by the stimulus. As depicted in Figure 1A, U87MG cells showed a notable increase in ADAM17 expression following TMZ treatment, with more than two-fold induction. In addition, we used an LDH assay to analyze the correlation between ADAM17 expression and TMZ sensitivity in GBM cell lines. DBTRG-05MG cells showed the highest resistance to TMZ, followed by U87MG cells, whereas M059K cells were the most sensitive to TMZ (Figure 1B). As shown in Figure 1C, DBTRG-05MG cells showed the highest transcript expression of ADAM17, followed by U87MG cells, whereas the lowest expression was observed in M059K cells, as assessed by qPCR. ADAM17 protein levels were detected by Western blotting, and the protein levels were consistent with transcript levels (Figure 1D). Next, we examined the effect of serum deprivation on ADAM17 expression in various GBM cell lines. As illustrated in Figure 1E–G, ADAM17 protein levels increased in a time-dependent manner in DBTRG-05MG, M059K, and U87MG cells after serum deprivation for 0–48 h. These results suggest that cells utilize ADAM17 expression as a cue of adapting to environmental changes, enhancing their resistance to adverse conditions and preventing cell death. Furthermore, we analyzed the expression levels of ADAM17 in the three GBM cell lines following TMZ treatment and observed a dose-dependent increase in ADAM17 expression at both the transcript (Figure 1H–J) and protein levels (Figure 1K–M) in all TMZ-treated GBM cell lines, indicating a possible link between ADAM17 levels and TMZ resistance in GBM.

### 2.2. ADAM17 Knockdown Restored the Chemosensitivity of TMZ-Resistant GBM Cells

**Figure 2:**
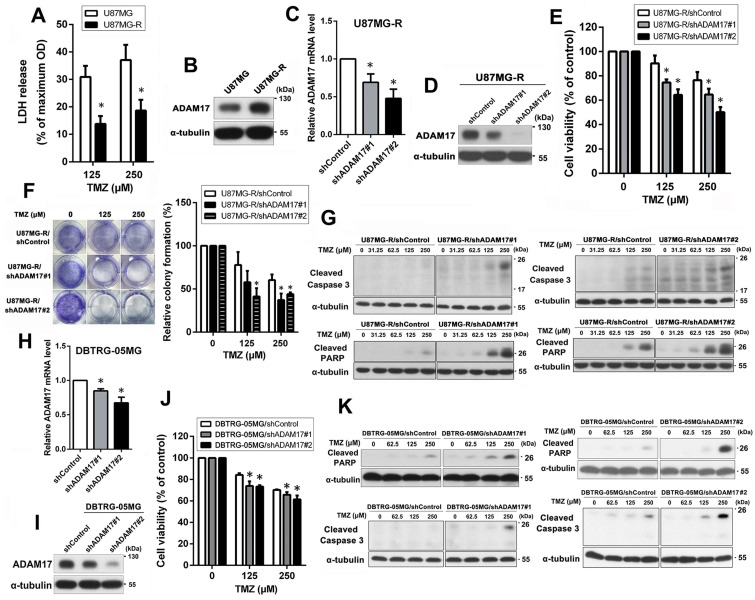
ADAM17 knockdown promoted the sensitivity of GBM cells to TMZ. (**A**) U87MG and U87MG-R cells were exposed to 125 or 250 μM of TMZ for 48 h. Cell death was evaluated using an LDH-based cytotoxicity assay. * *p <* 0.05, compared with U87MG. (**B**) ADAM17 protein expression in U87MG and U87MG-R cells was evaluated by Western blotting. (**C**,**D**) The efficiency of knockdown was analyzed using qPCR and Western blotting. (**E**) Cells were exposed to increasing doses of TMZ for 48 h. Subsequently, cytotoxicity was assessed using the MTT assay. * *p <* 0.05, compared with the shControl group. (**F**) Effect of ADAM17 knockdown on the TMZ sensitivity of U87MG-R cells was determined using colony formation assay. A representative image (left panel) and quantification (right panel) are shown. * *p <* 0.05, compared with the shControl group. (**G**) Cells were exposed to a range of TMZ doses (0–50 μM) for 48 h, and the expression levels of cleaved caspase-3 and cleaved PARP were analyzed using Western blotting. α-Tubulin was utilized as the loading control. (**H**,**I**) The efficiency of ADAM17 knockdown in DBTRG-05MG cells was confirmed using qPCR and Western blotting. (**J**) Cells were exposed to increasing concentrations of TMZ (0–250 μM) for 48 h, and cell viability was evaluated through the MTT assay. * *p <* 0.05, compared with the shControl group. (**K**) To evaluate the effect of ADAM17 knockdown on TMZ sensitivity in DBTRG-05MG cells, the levels of cleaved caspase-3 and cleaved PARP were measured using Western blotting. α-Tubulin was utilized as the loading control.

To further explore the role of ADAM17 in TMZ sensitivity of GBM cells, a TMZ-resistant U87MG subline was established, termed U87MG-R. First, we used an LDH assay to analyze the sensitivity of U87MG and U87MG-R cells to TMZ. U87MG-R cells treated with 125 and 250 μM TMZ showed significantly reduced cell death, with the percent LDH release of 13.78% and 18.63%, respectively. Meanwhile, U87MG cells released 30.89% and 37.07% LDH, respectively (Figure 2A). Therefore, U87MG-R cells exhibited greater TMZ resistance than the parental U87MG cells. Next, we examined ADAM17 protein expression in U87MG and U87MG-R cells using Western blotting. As shown in Figure 2B, ADAM17 protein levels in U87MG-R were significantly higher than in those in U87MG cells. To determine the effect of ADAM17 expression on TMZ sensitivity, ADAM17 was knocked down in U87MG-R cells using ADAM17 shRNA lentivirus transfection. The efficiency of ADAM17 knockdown was assessed using qPCR and Western blotting (Figure 2C,D). MTT (Figure 2E) and colony formation (Figure 2F) assays indicated that ADAM17 knockdown significantly increased TMZ sensitivity in a dose-dependent manner. In addition, we analyzed the expression levels of two canonical apoptosis-related molecules, PARP and caspase-3, using Western blotting to further confirm the effect of ADAM17 knockdown on TMZ sensitivity. As shown in Figure 2G, TMZ treatment markedly increased the levels of cleaved PARP and caspase-3 in ADAM17-knockdown U87MG-R cells compared with those in shControl cells. To demonstrate that ADAM17 is a common target related to TMZ sensitivity in GBM cell lines, gene knockdown was performed in another TMZ-lacking sensitive cell line, DBTRG-05MG. QPCR and Western blotting showed that the lentivirus-mediated knockdown system showed satisfactory efficiency of ADAM17 knockdown in DBTRG-05MG cells (Figure 2H,I). MTT assay revealed that ADAM17 silencing increased sensitivity to TMZ in a dose-dependent manner, as demonstrated in Figure 2J. Additionally, upon ADAM17 knockdown, TMZ-treated cells displayed a marked elevation in the levels of both cleaved PARP and cleaved caspase-3 compared with control cells (Figure 2K). Taken together, these results suggest that ADAM17 expression is associated with TMZ sensitivity in GBM cells.

### 2.3. miR-145-5p Regulates ADAM17 Expression by Targeting Its 3′-Untranslated Region (UTR) to Enhance TMZ Sensitivity in GBM Cells

**Figure 3:**
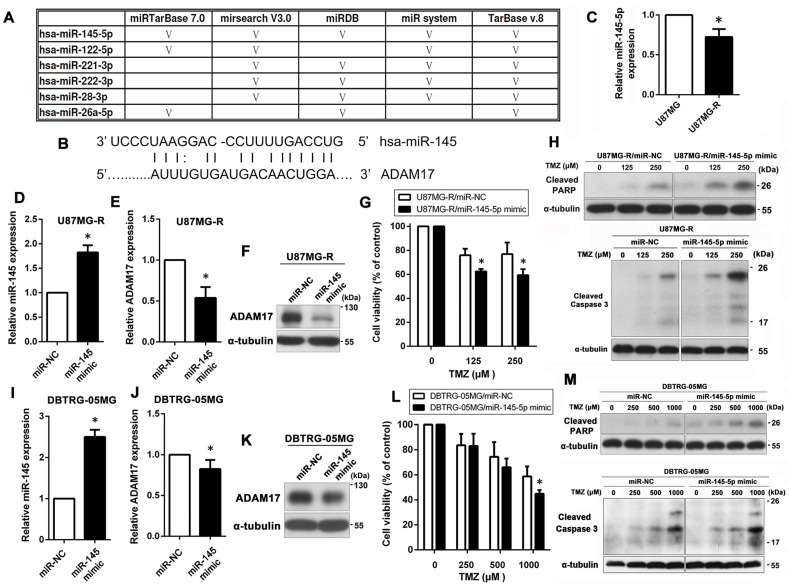
miR-145-5p targets ADAM17 to reduce its protein expression, leading to increased TMZ sensitivity in GBM cells. (**A**) Five bioinformatic algorithms were used to predict miRNAs that can potentially regulate ADAM17 expression. (**B**) The predicted target sequence of miR-145-5p in the 3′-UTR of ADAM17. (**C**) Relative expression levels of miR-145-5p were detected in resistant (U87MG-R) and parental (U87MG) cells using qPCR. * *p* < 0.05, compared with U87MG cells. (**D**–**H**) U87MG-R cells were transiently transfected with miR-145-5p mimics or negative controls (miR-NC), followed by TMZ treatment (0–250 μM) for 48 h. Subsequently, cytotoxicity was evaluated using the MTT assay and Western blotting. The levels of miR-145-5p and ADAM17 were validated using qPCR and Western blotting (**D**–**F**). Cell viability was determined using the MTT assay (**G**). Levels of cleaved PARP and cleaved caspase-3 were detected using Western blotting (**H**). (**I**–**M**) DBTRG-05MG cells were transiently transfected with miR-145-5p mimics or negative controls (miR-NC), followed by TMZ treatment (0–1000 μM) for 48 h. The effects of miR-145-5p mimic transfection on miR-145-5p and ADAM17 levels were validated using qPCR and Western blotting (**I**–**K**). Effects of transient transfection of miR-145-5p mimics on TMZ sensitivity were assessed using the MTT assay and Western blotting (**L**,**M**). α-Tubulin was used as the internal loading control. * *p* < 0.05, compared to miR-NC.

To further understand the molecular mechanism through which ADAM17 regulates TMZ resistance in GBM cells, we used five different miRNA-targeting prediction tools to identify upstream miRNA molecules that may potentially target ADAM17. All five databases showed that miR-145-5p targets ADAM17, most likely its upstream regulator (Figure 3A). The predicted target sites of miR-145-5p are shown in Figure 3B. Therefore, we hypothesized that ADAM17 is a target of miR-145-5p and that miR-145-5p downregulation in U87MG-R cells would elevate ADAM17 protein levels, thereby conferring TMZ resistance. As shown in Figure 3C, miR-145-5p levels were lower in U87MG-R cells than in U87MG cells. To further explore the effects of miR-145-5p on TMZ sensitivity, we used miR-145-5p mimics to increase intracellular levels of miR-145-5p in U87MG-R and DBTRG-05MG cells. We confirmed miR-145-5p overexpression using qPCR (Figure 3D,I) as well as ADAM17 transcript and protein expression using qPCR (Figure 3E,J) and Western blotting (Figure 3F,K), respectively. Subsequently, we used the MTT assay to analyze the effect of miR-145-5p on TMZ sensitivity in GBM cells. As shown in Figure 3G,L, miR-145-5p overexpression significantly enhanced TMZ cytotoxicity in U87MG-R and DBTRG-05MG cells. Similar results were obtained using Western blotting, which showed that the expression of cleaved PARP and cleaved caspase-3 in the miR-145-5p mimic group was markedly higher than that in the NC group following TMZ treatment (Figure 3H,M).

### 2.4. ADAM17 Expression Is Correlated to GBM Progression and Poor Prognosis

**Figure 4:**
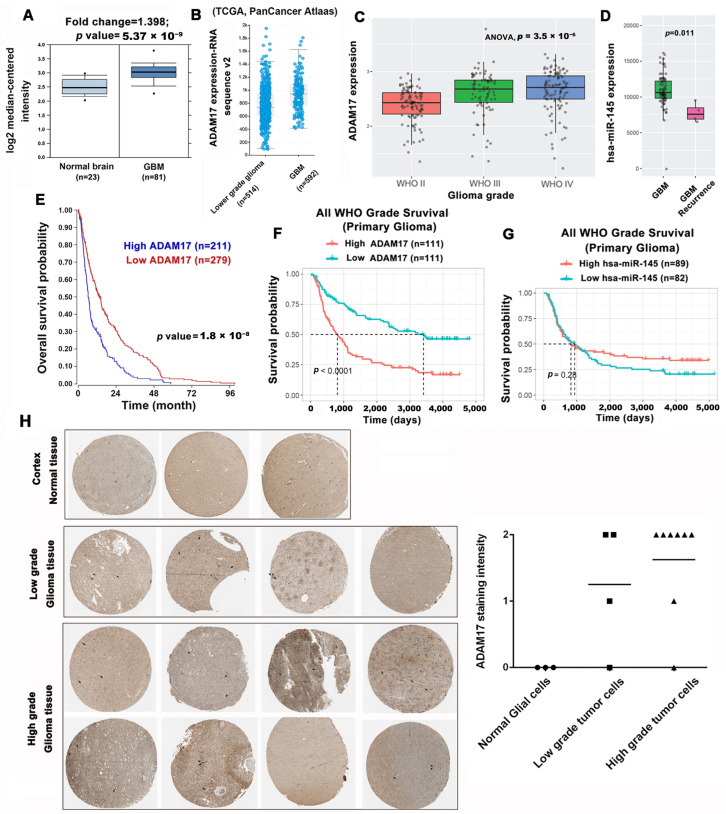
ADAM17 expression levels are associated with tumor progression and poor prognosis in GBM. (**A**) ADAM17 expression in normal brain and GBM tissues was analyzed using the Sun Brain dataset from Oncomine. (**B**) The levels of ADAM17 expression in low-grade glioma and GBM tissues were evaluated in the PanCancer Atlas dataset from cBioportal. Median and interquartile ranges are presented. (**C**) ADAM17 expression was positively correlated with tumor grade based on the CGGA database (mRNAseq_325 dataset). (**D**) The expression of miR-145 was downregulated in recurrent GBM tissues compared with that in primary GBM tissues, according to the analysis of data in the CGGA database (microRNA_array_198 dataset). (**E**) Clinical association of ADAM17 expression with the survival of patients with GBM as determined via Kaplan–Meier analysis. Data were obtained from the R2: Genomics Analysis and Visualization Platform database (Madhavan dataset). (**F**,**G**) The overall survival of patients with primary glioma presetting high or low expression of ADAM17 (mRNAseq_325 dataset) and miR-145 (microRNA_array_198 dataset), as analyzed using Kaplan–Meier survival analysis. (**H**) The Human Protein Atlas database shows the expression of ADAM17 protein in normal brain tissues form the cerebral cortex. ADAM17 expression levels at different histological grades were compared; the horizontal line represents the mean (definition: not detected = 0, weak = 1, and moderate = 2). Circles, squares, and triangles represent the scores of staining intensity in each group’s tissue.

To correlate ADAM17 expression with the clinical characteristics of GBM, we searched several publicly available databases. First, we analyzed the levels of ADAM17 using the Oncomine database to determine the differences in ADAM17 expression between GBM and normal brain tissues. As shown in Figure 4A, ADAM17 transcript expression was significantly higher (fold change = 1.398) in 81 GBM tissue samples than in 23 normal tissue samples (*p* = 5.37 × 10^−9^). In addition, based on TCGA data obtained through cBioPortal, we discovered that ADAM17 transcript levels were markedly elevated in GBM compared with those in low-grade glioma (Figure 4B). Furthermore, the expression pattern of ADAM17 in relation to GBM progression was investigated by analyzing RNA sequencing data obtained from the CGGA dataset, which were grouped according to the WHO glioma grades. ADAM17 expression increases with the pathological progression of glioma. The highest expression level was noted in grade IV gliomas, whereas the lowest expression level was noted in grade II gliomas (Figure 4C). Therefore, ADAM17 is a potential malignant biomarker for gliomas. Since miR-145 can target ADAM17 to downregulate its expression, we searched the CGGA database for the expression of miR-145 in primary and recurrent gliomas. As shown in Figure 4D, the expression level of miR-145 in recurrent GBM was lower than that in primary GBM. Next, we analyzed the prognostic value of ADAM17 using the R2 database and noted that increased expression of ADAM17 is significantly associated with poor overall survival (Figure 4E). Consistent results were obtained from the CGGA database, which demonstrated that higher ADAM17 expression may predict worse prognosis (Figure 4F). Conversely, patients with higher miR-145 expression showed better prognosis in the GCAA database (Figure 4G). In addition, we used data from the Human Protein Atlas to explore the association between ADAM17 protein expression in clinical specimens and disease progression. A marked increase in ADAM17 protein levels was noted in glioma tissues compared with those in normal brain tissue (Figure 4H). Collectively, our data show that ADAM17 levels are linked to the advancement of malignancy and inferior clinical outcomes in individuals diagnosed with glioma. Conversely, reduced miR-145 expression is correlated to poor prognosis and shortened survival.

### 2.5. ADAM17 Knockdown Enhanced TMZ Sensitivity In Vivo

**Figure 5:**
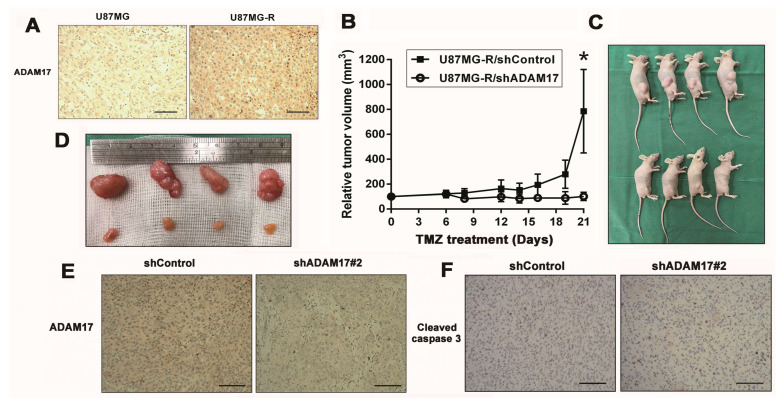
ADAM17 knockdown enhanced TMZ sensitivity of GBM xenograft tumors. (**A**) Immunohistochemical staining of ADAM17 in xenograft tumors derived from U87MG and U87MG-R cells. (**B**) Tumor growth curves of U87MG-R/shADAM17 and U87MG-R/shControl xenografts in mice following TMZ administration (n = 4). * *p <* 0.05, compared with the shControl group. (**C**) Images of subcutaneously injected xenograft tumors in nude mice at the end of the experiment. (**D**) Photographs of xenograft tumors from each group captured at the end of the experiment. (**E**,**F**) Representative images of IHC staining for ADAM17 and cleaved caspase-3 expression. Scale bar = 100 μm.

Finally, we subcutaneously inoculated nude mice with U87MG and U87MG-R cells and validated ADAM17 expression in both cell lines. As shown in Figure 5A, ADAM17 was highly expressed in U87MG-R cells compared with that in U87MG cells. Next, we inoculated ADAM17-knockdown and their control U87MG-R cells into nude mice. After subcutaneous tumor formation, each group was treated with TMZ for 21 days. The growth curve of xenografts showed that after ADAM17 knockdown, the transplanted tumor cells were more sensitive to TMZ, and the difference was statistically significant on day 19 after TMZ administration (Figure 5B). Mice bearing subcutaneous tumors were photographed at the end of the experiment to compare the effects of ADAM17 knockdown on tumor size (Figure 5C). To verify these findings, we extracted tumor tissues and visually assessed their size by imaging. Tumors in the ADAM17 knockdown group were significantly smaller than those in the control group after TMZ treatment, as demonstrated in Figure 5D. Tissues were further characterized by IHC. The expression of ADAM17 in the ADAM17 knockdown group was lower than that in the control group (Figure 5E). Moreover, cleaved caspase-3 levels in the ADAM17 knockdown group were significantly higher than those in the control group after TMZ treatment (Figure 5F). These in vivo data provide further evidence that ADAM17 silencing can sensitize tumor cells to TMZ, which is consistent with the in vitro findings.

## 3. Discussion

In the present study, we found that GBM cells induced ADAM17 expression when exposed to harsh environments (e.g., serum deprivation and TMZ treatment). As ADAM17 promotes the shedding of membrane-anchored proteins from the cell membrane, these secreted proteins can further bind receptors on the cell membrane, leading to the activation of the related signaling pathways. This implies that cancer cells overexpressing ADAM17 can exhibit elevated levels of secreted proteins, which may enhance their resistance to adverse external environments. Accordingly, we noted that ADAM17 was highly expressed in the established TMZ-resistant cell lines compared with that in the parental cell lines, which may have conferred TMZ resistance.

Ectodomain shedding is a process of proteolytic conversion in which a secreted transmembrane protein is released to modulate cellular functions [24,25,26]. Thus, ADAM17 plays a crucial role in various physiological and pathological processes, such as cell growth, regeneration, differentiation, inflammation, and cancer progression [10]. Furthermore, the primary cause of mortality in patients with cancer is not only resistance to treatment but also the spread of cancer cells to other parts of the body—a process called metastasis. A recent study has shown that ADAM17 inhibition can impair endothelial cell necroptosis and further prevent cancer metastasis, indicating the potential of this ADAM to serve as a potential target for the treatment of advanced-stage malignancies [27]. Similarly, in another study, we noted that ADAM17 knockdown inhibited cell invasion and metastasis in hepatocellular carcinoma [28].

ADAM17 has been reported to promote the malignant phenotype of glioma cells by increasing proliferation, invasion, angiogenesis, and tumor growth [29]. ADAM17 was upregulated in high-grade glioma tissues compared to that in low-grade and normal brain tissue from patients with glioma and, thus, has a diagnostic and prognostic value in patients with this malignancy [12]. Another study reported that ADAM17 levels were significantly elevated in the serum and ascites of patients with ovarian cancer, thus serving as a serum tumor marker for the early detection of this type of cancer [30]. Therefore, ADAM17 may be a potential blood biomarker for glioma staging.

Chemotherapeutic agents can activate ADAM17, leading to the cleavage of its substrates and acceleration of tumor growth. ADAM17 inhibition during chemotherapy may prove to be effective in combating drug resistance. A recent study on ovarian cancer treatment has shed light on this issue. As such, combination therapy with an ADAM17 inhibitor and cisplatin synergistically enhanced cancer cell apoptosis, and this approach holds promise for enhancing therapeutic efficacy in the future [31]. In addition, multiple studies have indicated that ADAM17 inhibition may represent a viable strategy for cancer treatment, making this ADAM a promising therapeutic target [32,33,34]. Moreover, ADAM17 inhibition has been reported to reduce the proliferation, migration, invasion, and differentiation of GBM cells [35]. According to a recent study, a monoclonal antibody against ADAM17, D8P1C1, effectively inhibited the proliferation of breast, ovarian, glioma, colon, and lung adenocarcinoma cells [36].

In addition to ADAM17 inhibitors, endogenous miRNAs can regulate ADAM17 protein levels; thus, miRNAs can be used in molecular diagnostic and therapeutic tools for GBM. Emerging research suggests that miRNAs are involved in the pathogenesis, progression, and therapeutic resistance of brain tumor [15,16,17]. In the present study, we identified miR-145-5p as an miRNA that may exert its effects by targeting ADAM17. Specifically, miR-145-5p overexpression enhanced the sensitivity of GBM cells to TMZ. In recent studies, miR-145 has been proven to inhibit the proliferation, invasion, and metastasis of tumor cells; increase the sensitivity of tumor cells to chemotherapy; and regulate the occurrence and development of tumors by targeting ADAM17 in several malignant tumors [23,37,38,39,40]. In particular, several studies have indicated that miR-145 could inhibit epithelial-to-mesenchymal transition [41], migration, and invasion [23] and increase the cytotoxicity of drug treatment in GBM [42,43]. Furthermore, the potential of miR-145 as a biomarker for predicting clinical outcomes of patients with GBM has been demonstrated [44,45]. In addition, other miRNAs that regulate ADAM17 expression have been explored. In gastric and hypopharyngeal cancer cells, miR-338-3p impedes cell migration and invasion by repressing ADAM17 expression [46,47]. Additionally, ADAM17 is highly expressed in drug-resistant colorectal cancer cells; however, miR-324-3p and miR-222 can suppress ADAM17 protein expression to restore drug sensitivity [48,49]. In hepatocellular carcinoma cells, miR-122 and miR-3163 upregulation can suppress ADAM17 levels, thereby inhibiting cell proliferation and enhancing sensitivity to antitumor drugs [50,51]. Furthermore, miR-224, miR-148a-3p, and miR-449b-3p can modulate ADAM17 expression in head and neck cancer to suppress malignancy [52,53,54]. Finally, miR-152 and miR-326 have been reported to inhibit cell proliferation, colony formation, migration, and invasion by silencing ADAM17 expression in lung cancer [55,56].

## 4. Materials and Methods

### 4.1. Cell Lines, Reagents, and Chemicals

The present study utilized three human GBM cell lines, namely DBTRG-05MG, M059K, and U87MG, which were obtained from the Bioresource Collection and Research Center (Food Industry Research and Development Institute, Hsinchu, Taiwan). Dulbecco’s modified Eagle’s medium (DMEM) with fetal bovine serum (FBS) (Thermo Fisher Scientific, Waltham, MA, USA) was used for cell culture. The primary antibody against ADAM17 for Western blotting (1:1000; cat. no. #3976) and cleaved caspase-3 (1:1000; cat. no. 9661) were obtained from Cell Signaling Technology Inc. Cleaved PARP (1:1000; cat. no. ab32064) was obtained from Abcam (Cambridge, UK). α-Tubulin (1:10,000; cat. no. 05-829) was obtained from EMD Millipore (Burlington, MA, USA). ADAM17 for immunohistochemistry (IHC) (1:750; cat. no. ab39162) was obtained from Abcam. To determine protein concentration, a BCA protein assay kit from Pierce (Appleton, WI, USA) (cat. no. 23235) was used. Additionally, the chemotherapeutic drug TMZ, solvent dimethyl sulfoxide (DMSO), and other necessary chemicals were obtained from Sigma-Aldrich Corp. (St. Louis, MO, USA).

### 4.2. Cell Culture and Drug Treatment

The cells were cultured in DMEM with 10% FBS, 100 units·mL^−1^ penicillin G, and 100 µg·mL^−1^ streptomycin in a 5% CO_2_ and 95% air environment at 37 °C. TMZ solutions were prepared in DMSO and subsequently diluted with the culture medium to ensure that the final concentration of DMSO did not exceed 0.05%.

### 4.3. RNA Sequencing

We used REzol reagent (Protech Technology Enterprise Co., Ltd., Taipei, Taiwan) to extract total RNA and submitted the samples to Genomics Co., Ltd. (New Taipei City, Taiwan) for library construction and sequencing. The NovaSeq 6000 sequencing platform (Illumina, San Diego, CA, USA) was used to generate 150 bp paired-end reads. To calculate the transcripts per million (TPM) for each gene, we used RSEM.

### 4.4. Lactate Dehydrogenase (LDH) Assay

The cytotoxicity of TMZ to GBM cells was evaluated by measuring the leakage of LDH into the extracellular fluid. A microplate reader was used to measure the absorbance of the supernatant from each sample at 490 nm. The release of LDH was expressed as the percentage of the total LDH activity (LDH in the medium plus LDH in the cells) using the following equation:

% LDH release = (LDH activity in the medium/total LDH activity) × 100.

### 4.5. Quantitative Real-Time PCR

RNA was extracted from the samples using TRIzol reagent (MD Bio Inc., Taipei, Taiwan), and reverse transcribed using the PrimeScript RT Reagent Kit (Perfect Real Time) from TaKaRa Bio Inc. (Otsu, Japan) to generate cDNA. qPCR was performed using the StepOnePlus Sequence Detection System (Applied Biosystems, Waltham, MA, USA). The thermal cycling protocol involved an initial activation step at 95 °C for 30 s, followed by a denaturation step at 95 °C for 2 min, and a final annealing and extension step at 60 °C for 30 s. This process was repeated for 40 cycles, according to the manufacturer’s protocol. qPCR was performed using the following primers: ADAM17: 5′-GTATCTGAACAACGACACCTG-3′ (forward) and 5′-CTCCTGGCACTTCTTCTGG-3′ (reverse); GAPDH: 5′-CACCCATGGCAAATTCCATGGCA-3′ (forward) and 5′-TCTAGACGGCAGGTCAGGTCCACC-3′ (reverse). The relative expression of genes was quantified using the comparative C_t_ (2^−∆∆CT^) method, with GAPDH serving as the normalization control.

### 4.6. Western Blotting

Cellular proteins were extracted using RIPA buffer containing a cocktail of protease inhibitors, and protein concentration was determined using a Pierce BCA protein assay kit. The extracted proteins were separated using SDS–PAGE and transferred onto PVDF membranes (Millipore, Bedford, MA, USA). The membranes were treated with a blocking buffer (5% nonfat milk) to decrease non-specific binding and then incubated overnight with specific primary antibodies at 4 °C. Next, the membranes were washed with Tris-buffered saline solution containing 0.05% Tween 20 and incubated with the appropriate peroxidase-conjugated secondary antibodies. After additional washing, the blots were detected using a chemiluminescent substrate (cat. no. 34580; Thermo Fisher Scientific) and analyzed using autoradiography.

### 4.7. Establishment of TMZ-Resistant Cells

A strain of U87MG-R cells that were able to withstand the effects of TMZ was developed by first exposing the parental cells to TMZ at a concentration of 15.625 μM and gradually increasing the TMZ concentration to 250 μM [57].

### 4.8. Lentiviral Systems for ADAM17 Knockdown

The lentiviral vectors used in the present study were obtained from the National RNAi Core Facility at the Academia Sinica (Taipei, Taiwan). These vectors are based on the pLKO.1-puro construct. To generate recombinant lentiviruses, 293T cells were transfected with a combination of 2 μg ADAM17 shRNA and packaging plasmids (2 μg pCMVΔR8.91 and 0.2 μg pMD) using a transfection reagent (PolyJet, SignaGen Laboratories, Frederick, MD, USA; cat. no. SL100688). The resulting lentiviral supernatant was then used to infect GBM cells in the presence of polybrene (8 μg·mL^−1^) for 24 h. Thereafter, the medium was replaced with fresh medium, and the cells were cultured for an additional 48 h. Stable cell lines were established by selecting cells that were resistant to puromycin (5 μg·mL^−1^). The lentiviral plasmids targeting ADAM17 were TRCN0000052172 (shADAM17#1: 5′-CCGGCCTATGTCGATGCTGAACAAACTCGAGTTTGTTCAGCATCGACATAGGTTTTTG-3′), TRCN0000052168 (shADAM17#2: 5′-CCGGCCAGCAGCATTCGGTAAGAAACTCGAGTTTCTTACCGAATGCTGCTGGTTTTTG-3′), and TRC2-pLKO_TRC001 (scrambled shControl).

### 4.9. MTT Assay

Cell viability was evaluated using the MTT assay. Specifically, 5000 cells were placed in each well of a 96-well plate containing 100 µL of medium and were left to adhere overnight. The medium was then replaced with serum-free DMEM, and the cells were incubated for 4 h before being exposed to varying concentrations of TMZ for 48 h. Prior to the end of the treatment period, the MTT reagent (0.5 mg·mL^−1^) was added to each well and incubated for 4 h. The medium was removed, and formazan crystals dissolved in 100 μL of DMSO were added. The absorbance of the dye was measured at 550 nm using an SpectraMax ABS Plus ELISA Microplate Readers (Molecular Devices, San Jose, CA, USA) and subtracted from the background value measured at 750 nm.

### 4.10. Colony Formation Assay

The cells were seeded in 6-well plates at the density of 5 × 10^4^ cells per well. Following incubation for 24 h to allow attachment, the cells were treated with an increasing concentrations of TMZ (ranging from 125 to 250 μM) for 48 h. The treated cells were harvested, re-plated in fresh 6-well plates containing 2 mL of medium, and incubated for an additional 2 weeks. Visible colonies were fixed in methanol, stained with crystal violet, imaged, and counted.

### 4.11. Bioinformatic Prediction of miRNAs Targeting ADAM17

We used five miRNA databases, namely miRTarBase 7.0 (http://mirtarbase.mbc.nctu.edu.tw/php/index.php accessed on 28 March 2023), mirsearch V3.0 (https://www.exiqon.com/mirsearch accessed on 28 March 2023), miRDB V5.0 (http://www.mirdb.org/ accessed on 28 March 2023), miR system (http://mirsystem.cgm.ntu.edu.tw/ accessed on 28 March 2023), and TarBase v.8 (http://carolina.imis.athena-innovation.gr/diana_tools/web/index.php?r=tarbasev8%2Findex accessed on 28 March 2023) to predict miRNAs targeting ADAM17. All experiments were accessed on 28 April 2019.

### 4.12. miRNA Expression Analysis

miRNA expression levels were detected via SYBR Green-based real-time PCR with the mir-X miRNA qRT-PCR SYBR kit (Clontech Laboratories, Inc., Palo Alto, CA, USA) according to the manufacturer’s protocol using 200–400 ng cDNA and 0.5 µL of 100 nM primers.

### 4.13. Cell Transfection with miRNA Mimics

To overexpress miR-145-5p, 25 nM of mirVana™ miRNA mimic or negative control was utilized. The GBM cell lines were transfected using TurboFect (Thermo Fisher Scientific) in accordance with the manufacturer’s guidelines.

### 4.14. Public Database Analysis

Oncomine (https://www.oncomine.org/ accessed on 28 March 2023) was used to analyze and compare the differences in ADAM17 transcript expression between human GBM and normal brain tissues. The transcript expression of ADAM17 in GBM and low-grade glioma cells was compared using cBioPortal (https://www.cbioportal.org/ accessed on 28 March 2023). In addition, the transcript expression of ADAM17 in grade II, grade III, and IV gliomas and expression level of miR-145 in primary and recurrent GBM tissues were analyzed using the Chinese Glioma Genome (CGGA) database (http://www.cgga.org.cn/n accessed on 28 March 2023). The association of ADAM17 transcript and miR-145 expression with clinical prognosis was analyzed using R2: Genomics Analysis and Visualization Platform (https://hgserver1.amc.nl/cgi-bin/r2/main.cgi accessed on 28 March 2023) and CGGA database, and data were presented as Kaplan–Meier curves. Data from R2 were extracted from the brain tumor dataset (Madhavan-550-MAS5.0-u133p2; 213532_at). The Human Protein Atlas (www.proteinatlas.org accessed on 28 March 2023) was used to analyze ADAM17 protein expression in clinical glioma and normal brain specimens, including three pairs of normal tissues, four pairs of low-grade glioma tissues, and six pairs of high-grade glioma tissues, using an ADAM17 antibody (HPA010738) for IHC [58]. All experiments were accessed on 8 October 2020.

### 4.15. Xenograft Mouse Model

Female BALB/c nude mice aged 4–6 weeks were acquired from BioLASCO (Taipei, Taiwan). These animals were housed in aseptic-lidded cages at the Animal Center of Chang Gung Memorial Hospital (Chiayi, Taiwan), which has been certified by the Association for Assessment and Accreditation of Laboratory Animal Care International (AAALAC International, Frederick, MD, USA). All experimental protocols were reviewed and approved by the Ethics Committee of Chang Gung Memorial Hospital (Chiayi, Taiwan) under the approval number 2019092602 with a valid period of 1 January 2020 to 30 June 2023. The mice were subcutaneously inoculated with 5 × 10^6^ cells on the right side. Tumor size was determined using calipers, and tumor volume was calculated using the following formula: length × width^2^ × 0.5. Once the tumors reached an approximate volume of 60 mm^3^, mice with xenografts were administered TMZ via intraperitoneal injection every 3 days for 21 days. At the end of the experiment, the mice were euthanized using excess CO_2_. The tumors were carefully removed via surgery from each mouse and fixed in 3.7% formaldehyde for IHC.

### 4.16. IHC

The fixed specimens were processed, embedded in paraffin, sliced into 4 μm thick sections, and baked at 65 °C for 30 min. The tumor sections were deparaffinized using xylene and then treated with 5% H_2_O_2_ for 20 min at room temperature to block endogenous peroxidase activity, followed by incubation with 3% BSA to prevent nonspecific binding. The sections were then incubated overnight at 4 °C with primary antibodies against ADAM17 (1:750) or cleaved caspase-3 (1:200). After washing, the sections were incubated with a biotinylated anti-rabbit secondary antibody for 1 h, followed by streptavidin for 1 h at room temperature. The sections were stained with DAB substrate chromogen solution and counterstained with hematoxylin.

### 4.17. Statistical Analysis

Data are presented as the mean of results obtained from at least three independent experiments and standard deviation (SD). To determine significant differences between groups, paired Student’s *t*-test was used. For comparison among multiple groups, one-way ANOVA was applied, followed by Tukey’s test. Results were considered statistically significant at *p* < 0.05.

## 5. Conclusions

Our findings suggest that ADAM17 levels can be increased by serum deprivation or TMZ treatment. ADAM17 can serve as a signaling scissor in the tumor microenvironment, enabling cancer cells to enhance their resistance to their surroundings. This inference was verified in our established TMZ-resistant cell lines, and the highly resistant cells showed high ADAM17 expression. Furthermore, bioinformatics revealed that elevated ADAM17 expression was negatively correlated with miR-145 levels, which was in turn correlated with tumor malignancy and poor survival. Both ADAM17 knockdown and miR-145 overexpression increased the sensitivity of GBM cells to TMZ. These findings provide a rationale for the development of novel diagnostic and therapeutic strategies for GBM.

## Data Availability

The data presented in this study are available within the article or upon reasonable request from the corresponding author.
